# Adverse Effects of Micro- and Nanoplastics on Humans and the Environment

**DOI:** 10.3390/ijms242115822

**Published:** 2023-10-31

**Authors:** Elena Niccolai, Ilaria Colzi, Amedeo Amedei

**Affiliations:** 1 Department of Experimental and Clinical Medicine, University of Florence, 50134 Florence, Italy; elena.niccolai@unifi.it; 2 Laboratorio Congiunto MIA-LAB (Microbiome-Immunity Axis Research for a Circular Health), University of Florence, 50134 Florence, Italy; 3 Department of Biology, University of Florence, 50121 Florence, Italy; ilaria.colzi@unifi.it

The pervasive pollution caused by nano- and microplastics (N/MPLs) is a pressing concern, and was exacerbated during the COVID-19 pandemic due to the substantial release of disposable Personal Protective Equipment (PPE) into the environment. Plastics, which are omnipresent due to human activities, undergo fragmentation into minuscule particles measuring less than 5 mm (MPLs) or less than 100 nm (NPLs) due to diverse biological, chemical, and physical factors. N/MPLs infiltrate various environments—soils, air, freshwaters, and oceans—even spreading to remote parts of the world, such as the Antarctic coasts, and have become a serious global pollution problem. Although marine matrices have garnered the most research interest, contamination in soil ecosystems may be four to twenty-three times more significant than that in the oceans [1], especially due to the extensive use of chemical fertilizers and plastic film mulching in agriculture. Common industrial contaminating plastics include polyethylene (PE), polypropylene (PP), polystyrene (PS), and polyvinyl chloride (PVC). Large amounts of N/MPLs accumulated in terrestrial ecosystems cause different degrees of damage to soil’s physical structure, chemical nutrients, and microbial activities, and ultimately, to the whole soil–plant system. Synthetic N/MPLs must be distinguished from other microparticles, such as carbon-like particles primarily derived from the incomplete combustion of organic matter (including fossil fuels, biomass, wood, and hydrocarbons), as well as inorganic particles originating through natural process like the weathering of rocks and minerals, soil erosion, and volcanic activity, or industrial processes such as the grinding or cutting of inorganic materials. It is important to note that these particles can have additional impacts on humans and the environment that may extend beyond the scope of this paper. The effect of N/MPL accumulation in soil on plants has received less research attention than that in marine environments, despite the fact that plants are fundamental primary producers, constitute the basis of the human diet, and regulate the global climate. Moreover, plants represent the starting point for the bioaccumulation of NPLs, which can be transferred through the food chain, eventually leading to human exposure. In contaminated terrestrial environments, the physiological and biochemical processes of plants can undergo widespread toxic effects, including oxidative damage, genotoxicity, and consequent plant growth reduction [2], which can lead to yield reduction and economic loss in agroecosystems. Moreover, NPLs can be taken up by plant roots and transported to aboveground tissues, exposing animals and humans to the risk of consuming contaminated food [2]. Additionally, due to their high surface area, N/MPLs can carry various chemicals and pathogens from the environment [3]. Given these potential risks, there is an urgent need for comprehensive research on the effects of MPLs on the environment and living organisms.

Globally, N/MPL contamination poses a serious risk to ecosystems and biodiversity; they could have adverse effects on organisms, enter the food chain, and eventually reach humans through ingestion, inhalation, and skin contact, leading to severe health problems.

The environmental, economic, and health impact of N/MPLs has parallels with the historical impact of asbestos. These particles accumulate in human tissues, potentially triggering chronic inflammatory responses and neoplastic transformations, similar to asbestos fibers.

This Special Issue encompasses seven papers that investigate the molecular mechanisms and potential impacts of N/MPLs in vivo (mice and marine animal models), and in vitro (human cell lines).

Investigating the effects of N/MPL in aquatic environments using marine models is highly relevant to understanding the broader implications of microplastic pollution. Every year, an estimated 4.8–12.7 million metric tons of plastic enters the oceans, representing a significant environmental challenge [4]. Primary sources of oceanic plastic pollution include coastal cities, shipping activities, and coastal landfills, where plastic debris undergoes continuous fragmentation into microplastics and even smaller nanoplastics [5]. The ingestion of N/MPLs has been observed in many marine organisms, suggesting their widespread infiltration into this ecosystem [6]. Microplastics have considerable impacts on marine life, include growth inhibition, increased oxidative stress, decreased feeding behavior, genotoxicity, neurotoxicity, and compromised reproductive capability [7]. Notably, these N/MPLs can serve as carriers of harmful anthropogenic pollutants, including persistent, bioaccumulative, and toxic substances (PBTs), such as polychlorinated biphenyls (PCBs) and dioxins, further increasing environmental and health risks [8]. Concomitantly, experiments conducted on cells and animals have yielded interesting insights into the biodistribution and toxic effects of N/MPLs on terrestrial organisms.

These minute plastic particles have the potential to disturb various bodily systems, including the digestive, respiratory, endocrine, reproductive, and immune systems. In the digestive system, microplastic ingestion may lead to inflammation or an altered intestinal microbiome, resulting in an imbalance between beneficial and harmful bacteria and potentially leading to symptoms like abdominal pain, bloating, and changes in bowel habits [9]. Additionally, the absorption of environmental toxins, such as heavy metals and polycyclic aromatic hydrocarbons carried by microplastics, can lead to gastrointestinal symptoms, including nausea, vomiting, and abdominal pain [10]. When inhaled, microplastics can induce oxidative stress in the airways and lungs, resulting in respiratory symptoms like coughing, sneezing, and shortness of breath, along with associated fatigue and dizziness due to decreased blood oxygen levels. Mitochondrial damage in human respiratory cells and the potential risk of chronic obstructive pulmonary disease have also been identified as possible consequences of inhaling microplastics [10,11]. The detrimental effects of MPL absorption through the skin due to extensive contact with plastic particles from sources like dust, cosmetics, sanitary pads, and liquid hand cleansers is an area of ongoing investigation [12,13]. In this regard, it was found that the acute exposure of vaginal keratinocytes to N/MPLs resulted in cell toxicity, affecting viability, apoptosis, gene expression, oxidative stress pathways, and miRNAs [14]. Moreover, chronic exposure to these particles caused lasting alterations in DNA methyltransferases and demethylases, potentially impacting epigenetic regulation, aging, inflammation, and even malignant transformations. These findings underscore the toxic effects of N/MPLs on human keratinocytes and underline the importance of comprehensively evaluating the plastics used in hygiene products, especially for women, such as disposable period products [14]. N/MPLs also pose a substantial risk to the endocrine system by interfering with hormone production, release, transport, metabolism, and elimination. This interference can lead to a range of endocrine disorders, including metabolic, developmental, and reproductive disorders such as infertility, miscarriage, and congenital malformations. In many cases, additives present in N/MPLs, like plasticizers and biocides, can disrupt the endocrine system and fertility, due to their immunotoxic effects [15]. MPLs’ presence in the human placenta raises concerns about fetal development [16,17]. Furthermore, research is ongoing to understand the potential impact of microplastics on the human immune system, and evidence has been found of chronic inflammation and homeostasis disturbances in animal studies and the activation of innate immunity in human lung cells [18,19]. Collectively, cellular and animal experiments have elucidated the role of microplastics in inducing oxidative stress, disrupting cellular structures, triggering inflammatory responses, lipid metabolism disturbances, gut microbiota dysbiosis, and neurotoxicity [20,21]. The studies included in this compilation offer valuable insights into the biodistribution and toxic effects of N/MPLs, and their findings underscore the critical influence of particle size, polymer composition, and exposure duration in determining the extent of damage caused by N/MPLs. N/MPLs encompass a wide array of polymer types, sizes, and forms, making it challenging to generalize findings to all microplastic variants. The varying dosages employed across different studies further contribute to this complexity, underscoring the need for standardized exposure levels to conduct meaningful comparisons and draw insightful conclusions.

The complexities of environmental conditions and the multitude of sources contributing to microplastic pollution amplify the challenge of better defining microplastics’ effects. Real-world scenarios involve the complex interplay of microplastics with other pollutants, environmental factors, and biological responses, which are challenging to replicate accurately in a controlled laboratory setting. To address these limitations and foster a comprehensive understanding of N/MPLs’ effects, future studies should strive to establish standardized methodologies and diverse yet complementary models, and to conduct interdisciplinary collaboration.

In conclusion, the pervasiveness and harmful impacts of micro- and nanoplastics across diverse ecosystems demand urgent attention. Furthermore, the presence of additives in N/MPLs exacerbates their potential health risks, affecting endocrine systems and fertility and potentially contributing to neoplastic transformations. These findings have brought to light the relevance of the chemical identity of polymers, revealing potential antagonistic effects and the modulation of toxicity levels. This underscores the urgent need for research to encompass a wider range of pollutants and microplastics in order to comprehensively assess their combined impacts. By working together to bridge the gaps in our knowledge, we can enhance our understanding of the intricate dynamics of micro- and nanoplastic pollution and its potential impacts on both ecosystems and human health.
